# Importance of TKI treatment duration in treatment-free remission of chronic myeloid leukemia: results of the D-FREE study

**DOI:** 10.1007/s12185-023-03549-3

**Published:** 2023-02-04

**Authors:** Chikashi Yoshida, Hiroki Yamaguchi, Noriko Doki, Kazunori Murai, Masaki Iino, Yoshihiro Hatta, Makoto Onizuka, Norio Yokose, Katsumichi Fujimaki, Masao Hagihara, Gaku Oshikawa, Kayoko Murayama, Takashi Kumagai, Shinya Kimura, Yuho Najima, Noriyoshi Iriyama, Ikuyo Tsutsumi, Koji Oba, Hiroshi Kojima, Hisashi Sakamaki, Koiti Inokuchi, the Kanto CML Study Group

**Affiliations:** 1 Department of Hematology, National Hospital Organization Mito Medical Center, 280 Sakuranosato, Ibarakimachi, Higashiibarakigun, Ibaraki 311-3193 Japan; 2 Department of Hematology, Nippon Medical School, Tokyo, Japan; 3 Hematology Division, Tokyo Metropolitan Cancer and Infectious Diseases Center, Komagome Hospital, Tokyo, Japan; 4 Department of Hematology, Iwate Prefectural Central Hospital, Morioka, Japan; 5 Department of Medical Oncology, Yamanashi Prefectural Central Hospital, Kofu, Japan; 6 Division of Hematology and Rheumatology, Nihon University School of Medicine, Tokyo, Japan; 7 Department of Hematology and Oncology, Tokai University School of Medicine, Isehara, Japan; 8 Department of Hematology, Nippon Medical School Chiba Hokusoh Hospital, Inzai, Japan; 9 Department of Hematology, Fujisawa City Hospital, Fujisawa, Japan; 10 Department of Hematology, EIJU General Hospital, Taito-Ku, Japan; 11 Japanese Red Cross Musashino Hospital, Musashino, Japan; 12 Division of Hematology, Gunma Prefectural Cancer Center, Ohta, Japan; 13 Department of Hematology, Ome Municipal General Hospital, Ome-Shi, Tokyo Japan; 14 Division of Hematology, Respiratory Medicine and Oncology, Department of Internal Medicine, Faculty of Medicine, Saga University, Saga, Japan; 15 Department of Biostatistics, The University of Tokyo, Tokyo, Japan; 16 Ibaraki Clinical Education and Training Center, University of Tsukuba Hospital, Kasama, Japan

**Keywords:** Chronic myeloid leukemia, Dasatinib, Treatment-free remission

## Abstract

Treatment-free remission (TFR) is a new goal for patients with chronic myeloid leukemia in chronic phase (CML-CP) with a sustained deep molecular response (DMR) to treatment with tyrosine kinase inhibitors (TKIs). However, optimal conditions for successful TFR in patients treated with second-generation (2G)-TKIs are not fully defined. In this D-FREE study, treatment discontinuation was attempted in newly diagnosed CML-CP patients treated with the 2G-TKI dasatinib who achieved BCR-ABL1 levels of ≤ 0.0032% (MR4.5) on the international scale (*BCR-ABL1*^*IS*^) and maintained these levels for exactly 1 year. Of the 173 patients who received dasatinib induction therapy for up to 2 years, 123 completed and 60 (48.8%) reached MR 4.5. Among the first 21 patients who maintained MR4.5 for 1 year and discontinued dasatinib, 17 experienced molecular relapse defined as loss of major molecular response (*BCR-ABL1*^*IS*^ > 0.1%) confirmed once, or loss of MR4 (*BCR-ABL1*^*IS*^ > 0.01%) confirmed on 2 consecutive assessments. The estimated molecular relapse-free survival rate was 16.7% at 12 months. This study was prematurely terminated according to the protocol’s safety monitoring criteria. The conclusion was that sustained DMR for just 1 year is insufficient for TFR in CML-CP patients receiving dasatinib for less than a total of 3 years of treatment.

## Introduction

Prognosis of chronic myeloid leukemia in chronic phase (CML-CP) has improved dramatically since the approval of imatinib mesylate, the first-generation tyrosine kinase inhibitor (TKI), in 2001 [1]. In addition, second-generation TKIs (2G-TKIs) were developed for patients who are resistant and/or intolerant to imatinib and later approved for newly diagnosed patients [2–4]. With the development of these TKIs, the life expectancy of patients with CML-CP has become almost the same as that of age-matched healthy individuals [5]. However, long-term treatment with TKIs has raised new concerns, such as adverse events including cardiovascular side effects, avoidance of pregnancy by women due to possible teratogenicity, and increased health care costs [6–8]. Thus, discontinuation of TKI therapy has been attempted in patients with a sustained deep molecular response (DMR), and it has been reported that about half of them can maintain a treatment-free remission (TFR), which has become a new goal [9]. However, it is still difficult to predict in advance the chance of relapse for each patient. Although several guidelines have proposed clinical factors for successful TFR, they are primarily based on evidence with imatinib [10–12]. Since 2G-TKIs induce a molecular response faster than imatinib [2–4], it is possible that they lead to TFR in a larger number of patients and after a shorter treatment period, potentially minimizing the problems caused by TKI therapy. The multicenter phase II study D-FREE was conducted to clarify optimal conditions for TFR in newly diagnosed patients with CML-CP treated with the 2G-TKI dasatinib. We attempted discontinuation of dasatinib treatment for patients who achieved DMR and sustained it for exactly 1 year.

## Materials and methods

### Patients

Eligible patients were adults (≥ 18 years) with confirmed newly diagnosed CML-CP who had received no prior antileukemia treatment (except ≤ 1 months of hydroxyurea), Eastern Cooperative Oncology Group (ECOG) performance status groups of 0 to 2, and with adequate functions of major organs (liver, kidney, and lung). Patients whose *BCR-ABL1* mRNA levels could not be assessed by real-time quantitative polymerase chain reaction (RQ-PCR) based on an international scale (*BCR-ABL1*^IS^) were excluded. Patients were excluded if they had active multiple cancers, were pregnant, lactating, with a history or complications of myocardial infarction within the previous 6 months, with a history of angina pectoris, or gastrointestinal hemorrhage, or congestive cardiac failure within the previous 3 months, plural effusion, electrocardiogram QTc interval exceeding 450 ms prolongation, or present/past history of pulmonary hypertension. Similarly, patients with a history or complications of diseases judged by the investigators as inappropriate for study implementation were not eligible. This study was conducted in accordance with the Ethical Guidelines for Medical and Health Research Involving Human Subjects, the Clinical Trials Act in Japan, and the Declaration of Helsinki. All patients provided written informed consent. The study protocol was reviewed by the institutional review board for each center at the start of study, then reviewed again by the Certified Review Board, Institutional Review Board of Nippon Medical School Foundation, after the enforcement of the Clinical Trials Act established in 2018. The trial was registered at the University Hospital Medical Information Network (UMIN000022254) and Japan Registry of Clinical Trails (jRCTs031180332).

### Study design and treatment

**Fig. 1:**
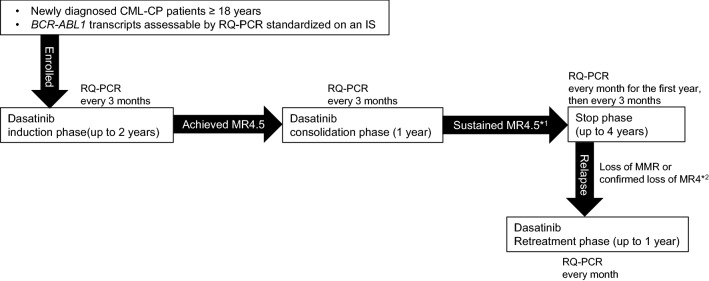
D-FREE study design. *CML-CP* chronic myeloid leukemia in chronic phase, *RQ-PCR* real-time quantitative polymerase chain reaction, *IS* international scale, *MR4.5 BCR-ABL1* mRNA levels assessed by RQ-PCR based on an IS (*BCR-ABL1*^IS^) ≤ 0.0032%), *MMR* major molecular response. *^1^ Defined as maintaining MR 4.5 with 5 RQ-PCR assessments every 3 months. *^2^ Defined as *BCR-ABL1*^*IS*^ > 0.01% on two consecutive assessments

D-FREE was an open-label, multicenter, phase II study. Its design is shown in Fig. 1. In the induction phase, newly diagnosed CML-CP patients were treated with dasatinib at 100 mg once daily. The maximum daily dose was 140 mg, and the dose could be adjusted or temporarily stopped at the discretion of investigators as needed. Molecular response was assessed by measuring *BCR-ABL1* mRNA levels in peripheral blood every 3 months by RQ-PCR standardized on an international scale in one of three commercial laboratories (BML, Inc., SRL, and LSI Medience Corporation) using an RT-qPCR kit, ODK-1201 (Otsuka Pharmaceutical Co. Ltd) validated by SA Pathology, Adelaide, Australia, a recognized reference laboratory [13]. When patients achieved MR4.5 (*BCR-ABL1*^IS^ ≤ 0.0032%) during the induction phase for up to two years, they were immediately entered into the consolidation phase, where dasatinib was administered for 12 months. Sustained MR4.5 in this phase was defined as confirmation of this level of molecular response on 5 consecutive RQ-PCR tests three months apart. Patients who did not achieve MR4.5 during the induction phase or did not sustain it during the consolidation phase were removed from the study and its follow-up. Patients who sustained MR.4.5 throughout the consolidation phase were eligible to enter the stop phase and discontinue dasatinib treatment. During the stop phase, molecular response was assessed every month in the first year and every three months thereafter. Molecular relapse was defined as loss of major molecular response (MMR) (*BCR-ABL1*^IS^ > 0.1%) confirmed once, or loss of MR4 (*BCR-ABL1*^IS^ > 0.01%) confirmed on 2 consecutive assessments. Currently, most recent trials consider molecular relapse as loss of MMR [9, 14], but since this D-FREE was a study in which dasatinib was discontinued after a shorter DMR period, the loss of MR4 confirmed on 2 consecutive assessments was also considered as molecular relapse for safety reasons. In the case of molecular relapse during the stop phase, patients were immediately retreated with dasatinib at the consolidation phase final dose. Molecular response was assessed every month by RQ-PCR until patients regained MR4 (*BCR-ABL1*^IS^ ≤ 0.01%), and every three months thereafter, for up to one year. Adverse events were graded according to the National Cancer Institute Common Terminology Criteria for Adverse Events (CTCAE) version 4.0. In this study, hematologic adverse events with grade 4 and non-hematological adverse events with grade 3 or higher were reported.

### Study endpoints and assessments

The primary endpoint was the proportion of patients in TFR who showed no molecular relapse and did not need to resume dasatinib 12 months after treatment discontinuation. Secondary endpoints included the proportion of patients in TFR 24, 36, and 48 months after discontinuation of dasatinib treatment, molecular relapse-free survival (MRFS) 12 months after discontinuation of dasatinib treatment, overall survival (OS) including all causes of death 12, 24, 36, and 48 months after discontinuation of dasatinib treatment, dasatinib doses and time to MR4.5, event-free survival (EFS) during the dasatinib treatment period, and frequency and degree of TKI withdrawal syndrome after discontinuation of dasatinib treatment. Exploratory endpoints included analysis of factors related to achievement of MR4.5 and sustained TFR for risk scores (EUTOS Score, Sokal Score, Hasford Score), gender, molecular response 3 and 6 months after start of dasatinib treatment, dasatinib treatment period, and time to MMR, MR4.0, and MR4.5. MRFS was defined as duration of survival from the date of dasatinib discontinuation in the stop phase to molecular relapse or death. EFS was defined as the duration from the date of registration to disease progress or death. If patients died without disease progress, they were regarded as having progress at the date of death. If patients received dasatinib and did not have disease progression or death, the study was discontinued at the date when hematologic, cytogenetic, or molecular evaluation was finally done during dasatinib treatment. If patients did not receive dasatinib and did not have disease progression or death, the study was discontinued at the date of registration. Disease progression was defined as loss of complete hematologic response, loss of major cytogenetic response or complete cytogenetic response, loss of molecular response, progression to accelerated or blastic phase, or death during dasatinib treatment.

### Statistical analyses

The threshold value of TFR rate and expected value at 12 months after discontinuation of dasatinib treatment were hypothesized to be 41% and 55%, respectively, based on historical data [15, 16]. Based on the above hypothesis, the required number of patients eligible for treatment discontinuation (Stop phase) was calculated to be 83 with a power of 80% and a one-sided alpha of 5%. Considering about 20% dropouts due to MR4.5 loss during the 12-month consolidation phase and consent withdrawal, etc., and the historical data of a 35% MR4.5 achievement rate after 24 months [17], the planned number of registered patients with first-onset CML-CP was calculated as 300, including 5% ineligible patients.

Two safety monitoring criteria were included in D-FREE to assess the appropriateness of continuing the study. Thus, the trial should be suspended if (1) more than 15% of the patients who lost MMR in the Stop phase did not reach MMR by 12 months of dasatinib retreatment and (2) the TFR rate was less than or equal to 25% when 20 patients reached the primary endpoint. On the 6th November 2019, 15 patients out of 20 patients after discontinuation of dasatinib treatment relapsed during the stop phase. Data and safety monitoring board recommended stopping the study on the basis of the efficacy concerns. When the study was stopped on the 3rd December 2019, 17 out of 21 patients in the stop phase relapsed. Thereafter, all participant patients were recommended to receive standard treatment based on consultation with their physicians. Baseline characteristics, efficacy, and safety results are reported for patients who entered the induction phase. MRFS was depicted using the Kaplan–Meier method. Other time-to-event data were also analyzed by the Kaplan–Meier method. An assessment of prognostic factors for achieving MR4.5 within 24 months of dasatinib treatment in the induction phase was conducted using univariate and multivariable logistic regression analyses. The frequencies of AEs, laboratory abnormalities, and predefined groupings of AE types of special interest were summarized for the induction and consolidation phases. The data presented herein are based on a cutoff date of September 30th, 2021, at which time all patients had finished the safety follow-up after the termination of study treatment. The significance level of the two-sided p values was 0.05 for all statistical tests. Analyses were done with SAS Release 9.4 (SAS Institute, Cary, NC).

## Results

### Characteristics of patients

**Fig. 2:**
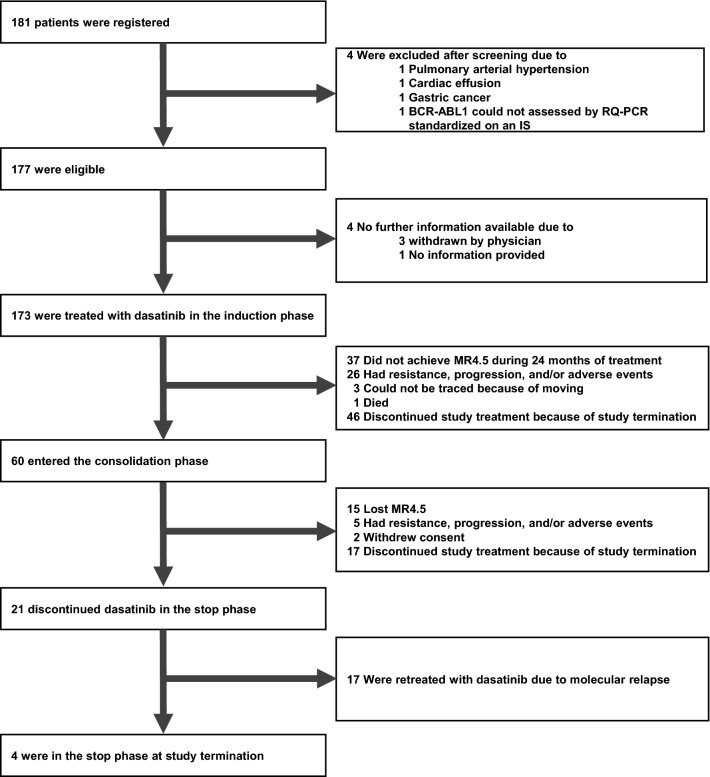
Patient disposition. *RQ-PCR* real-time quantitative polymerase chain reaction, *IS* international scale, *MR4.5 BCR-ABL1* mRNA levels assessed by RQ-PCR based on an IS ≤ 0.0032%)

Between July 2016 and May 2019, a total of 181 patients with newly diagnosed CML-CP were enrolled in 41 centers in Japan. Patients’ disposition is shown in Fig. 2. Four patients were excluded after screening, and no further information was available for 4 patients. Overall, 173 patients received treatment according to the study protocol. Demographics and baseline disease characteristics are listed in Table1. The median patient age was 54 years (18–83 years). The rates of Sokal low-, intermediate-, high-risk groups, and unknown were 45.7, 38.2, 15.6, and 0.6%, respectively. The rates of Hasford low-, intermediate-, high-risk groups, and unknown were 39.9, 50.9, 8.7, and 0.6%, respectively. The rates of EUTOS low- and high-risk groups were 89.6 and 10.4%, respectively.

**Table 1:** Baseline characteristics at study entry

Characteristic	Total population (*n* = 173)
Age, median (range) (years)	54 (18–83)
Males, *n* (%)	100 (57.8)
Sokal risk score, *n* (%)	
Low	79 (45.7)
Intermediate	66 (38.2)
High	27 (15.6)
NR	1 (0.6)
Hasford risk score, *n* (%)	
Low	69 (39.9)
Intermediate	88 (50.9)
High	15 (8.7)
NR	1 (0.6)
EUTOS risk score, *n* (%)	
Low	155 (89.6)
High	18 (10.4)
Prior hydroxyurea treatment, *n* (%)	
No	124 (71.7)
Yes	49 (28.3)

*NR* not reported

### Treatment responses

Of the 123 patients who completed the induction phase, 60 (48.8%) achieved MR4.5 for up to 2 years. The median duration of dasatinib for achieving MR4.5 was 7.7 months (range 3.0–21.1 months). We evaluated the relationship between cumulative dose and MR4.5 attainment rate and calculated the cumulative dose corresponding to MR4.5 attainment rate of 50% (for one subject there was no dose information). The total dasatinib dose corresponding to 50% MR4.5 by the Kaplan–Meier method was 63,600 mg (95% confidence interval 43,500 to N.A.), but the 95% confidence interval was wide due to the small number of patients who reached MR4.5, and the upper limit of the 95% confidence interval could not be estimated (data now shown). Single and multivariate analyses showed that the achievement of MMR at 3 months, but not gender, Sokal risk score, Hasford risk score, EUTOS risk score, or age (< 60 vs. ≥ 60), was predictive of the achievement of MR4.5 within 2 years (Table 2).

**Table 2:** Prognostic factors for achieving MR4.5 within 24 months of dasatinib treatment in the induction phase

	Univariate			Adjusted		
	Odds ratio	95% CI	*p*	Odds ratio	95% CI	*p*
Gender						
Male	0.853	0.458–1.589	0.853	0.768	0.370–1.596	0.4797
Female	1			1		
Sokal risk score						
High	1.133	0.456–2.812	0.7879	1.466	0.451–4.767	0.5253
Intermediate	1.419	0.723–2.785	0.3088	1.002	0.404–2.484	0.9966
Low	1			1		
Hasford risk score						
High	1.25	0.398–3.931	0.7026			
Intermediate	1.238	0.644–2.381	0.5219			
Low	1					
EUTOS score						
High	0.814	0.290–2.284	0.6952	0.604	0.153–2.384	0.4715
Low	1					
Age, years						
60 ≥	1.55	0.830–2.893	0.1687	1.531	0.668–3.512	0.3144
60 <	1			1		
MMR at 3 months						
Yes	9.412	4.058–21.831	< 0.0001	11.192	4.583–27.334	< 0.0001
No	1			1		

Patients who achieved MR 4.5 in the induction phase immediately entered the consolidation phase and received dasatinib treatment for one year. Fifteen patients could not sustain MR4.5 and finished study treatment during the consolidation phase. EFS for patients treated with dasatinib during the induction and consolidation phases was estimated to be 94.9% for 1 year and 63.5% for 2 years. A large number of patients were censored due to early discontinuation of the study, as described above.

### Discontinuation of dasatinib in the stop phase

**Fig. 3:**
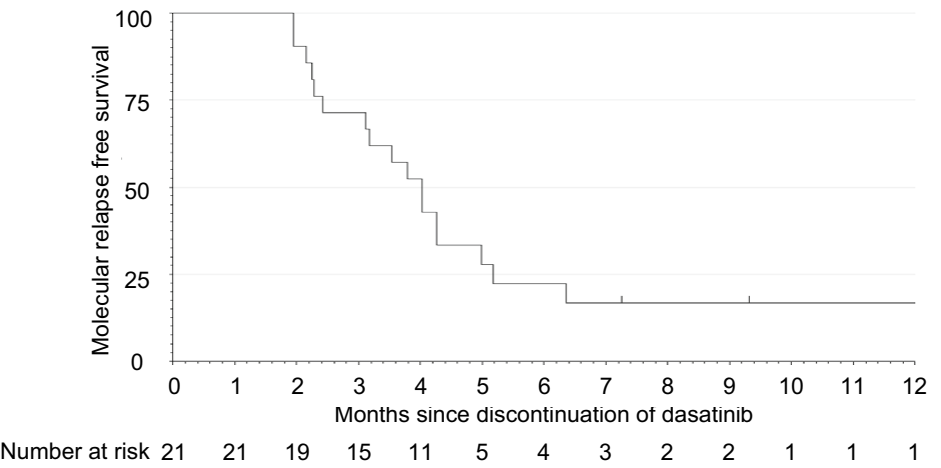
Kaplan–Meier estimate of molecular relapse-free survival after discontinuation of dasatinib in the first 21 patients who entered the stop phase

**Fig. 4:**
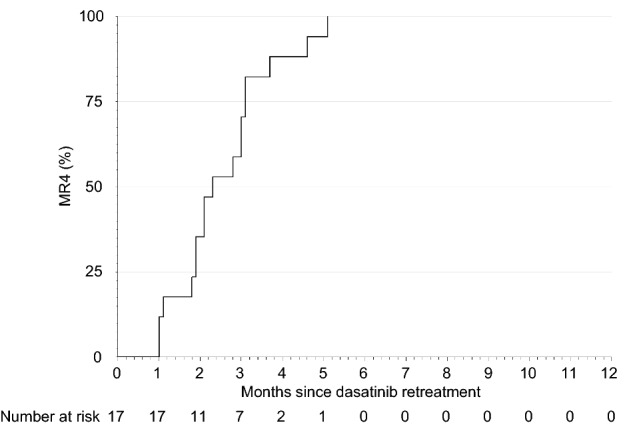
Cumulative incidence of MR4 in patients who entered the dasatinib retreatment phase due to molecular relapse. *MR4 BCR-ABL1* mRNA levels assessed by RQ-PCR based on an international scale ≤ 0.01%

Among the first 20 patients who could sustain MR4.5 for 12 months in the consolidation phase and discontinued dasatinib treatment in the stop phase, 15 experienced molecular relapse within 12 months and required retreatment with dasatinib as of November 6, 2019. The study was terminated prematurely on December 3, 2019, in accordance with the data and safety monitoring board’s recommendation, based on the pre-specified interim analysis criterion that it would be stopped if the TFR rate was less than or equal to 25% in the first 20 patients of the stop phase. At that point in time, 17 out of 21 patients in the stop phase had molecular relapse within 12 months and were being retreated with dasatinib (Table 3). Molecular relapse was determined by the loss of MMR in 11 patients and two consecutive losses of MR4 in 6 patients. The estimated MRFS was 16.7% at 12 months (Fig. 3). As a result, the proportion of patients in TFR who showed no molecular relapse and did not need to resume dasatinib 12 months after treatment discontinuation was not estimated, because only one subject was confirmed to have TFR at 12 months after discontinuation of dasatinib. The median time of molecular relapse after the cessation was 3.5 months (range 2.0–6.4). In those patients, the median duration of dasatinib treatment, including the induction and the consolidation phases, was 18.9 months (range 14.9–25.5) before the cessation of dasatinib. At termination, 46 patients were in the induction, 17 were in the consolidation, and 4 were in the stop phase. Of note, one patient (no. 7) remained in TFR for 17.8 months after receiving dasatinib treatment for only 18.6 months during the induction and consolidation phases (Table 3). All 17 patients who entered the dasatinib retreatment phase due to molecular relapse regained MR4 (Table 3 and Fig. 4). The median duration of dasatinib retreatment until MR4 re-achievement was 2.3 months (range 1.0–5.1).

**Table 3:** Details of patients in the stop phase

Pt no.	Age, y	Gender	Sokal risk score	Hasford risk score	EUTOS score	Duration of induction phase, mo	Duration of DAS treatment, mo	Duration of stop phase, mo	Relapse after DAS discontinuation	Achievement of MR4 after DAS retreatment, mo
1	77	F	H	H	L	3.1	15.7	6.3	Yes	1.9
2	72	F	H	H	H	3.4	14.9	3.2	Yes	3.7
3	68	M	H	I	H	13.3	25.5	2.5	Yes	2.8*^4^
4	62	M	I	I	L	6.0	18.9	2.4	Yes	3.1
5	64	F	I	I	L	8.9	22.0	3.4	Yes	1.0
6	38	M	L	L	L	6.4	18.9	4.8	Yes	5.1
7	53	F	H	I	L	5.8	18.6	17.8	No	–
8	72	F	I	I	L	6.5	19.4	2.5	Yes	2.1
9	77	F	I	I	L	6.3	18.3	5.0	Yes	1.0
10	49	F	I	L	L	12.2	24.8	3.9	Yes	3.0
11	57	M	L	I	H	8.7	21.7	3.8	Yes	2.1
12	29	F	I	L	L	6.0	18.6	4.4	Yes	3.0
13	63	F	I	I	L	3.5	16.2	2.3	Yes	1.8
14	68	F	I	I	L	6.3	19.2	9.2	No	–
15	64	F	I	I	L	8.8	21.6	4.8	Yes	2.3
16	38	F	L	L	L	5.7	18.8	5.3	Yes	1.1
17	69	F	I	I	L	6.0	18.9	7.1	No	–
18	54	M	L	I	L	6.0	18.7	4.6	Yes	1.9
19	27	M	L	L	L	5.8	17.8	4.1	Yes	4.6
20	70	M	I	L	L	6.2	18.6	4.4	No	–
21	46	M	L	L	H	6.9	18.9	2.6	Yes	3.1

*Pt* patient, *no.* number, *y.* years, *F* female, *M* male, *L* low risk, *I* intermediate risk, *H* high risk, *mo.* Months, *DAS* dasatinib, —, not applicable

*^4^Reached MR4 after 2.8 months of DAS retreatment phase and simultaneously discontinued study treatment due to adverse events

### Safety

No patients progressed to the accelerated/blastic phase or died due to CML during the study treatment. Grade 4 hematologic and grade 3/4 non-hematologic AEs were reported in this study. Among patients who were treated with dasatinib in the induction phase, grade 4 neutropenia, anemia, and thrombocytopenia were reported in 2.3% (*n* = 4), 0.6% (*n* = 1), and 1.2% (*n* = 2), respectively. There were no grade 4 hematologic AEs reported in consolidation, stop, and dasatinib retreatment phases. Grade 3/4 non-hematologic AEs were reported as a total of 8.1% (*n* = 14) in the combined induction, consolidation, and retreatment phases. Among them, death in one patient (unknown cause; family refused to report) and hospitalization due to gallstones, both occurring in the induction phase, were reported as grade 4 AEs. Cardiovascular events were reported in 2 patients (1.2%), one with grade 3 ischemic syndrome during the consolidation phase and the other with grade 3 ischemic cerebrovascular disease during the dasatinib retreatment phase. Among the 21 patients who entered the stop phase, TKI withdrawal syndrome was observed in 2 (no. 1 and no. 20, Table 3). Two patients (9.5%) had arthralgia grade 1 and one (4.8%) had myalgia grade 1, both of whom recovered. The two patients who developed TKI withdrawal syndrome both had molecular relapse at 2.6 and 6.3 months.

## Discussion

Discontinuation of treatment in newly diagnosed CML-CP patients after up to 2 years of induction therapy with dasatinib followed by 1 year of MR4.5 did not show TFR rates comparable to previous TKI discontinuation studies, which have reported rates of approximately 50% [18–21]. This indicates that dasatinib treatment for 3 years or less, including just 1 year of MR4.5 maintenance, is not sufficient for successful TFR in untreated patients with CML-CP.

2G-TKIs, including dasatinib and nilotinib, are stronger inhibitors of ABL kinase activity than imatinib and have been the subject of several clinical trials because of their potential to improve TFR rates. Comparisons of TKI discontinuation trials in patients who received 2G-TKI as first-line treatment, including D-FREE study, are summarized in Table 4. The ENESTfreedom study enrolled patients who were treated with nilotinib ≥ 2 years and reached MR4.5 [20]. Those patients were treated with nilotinib for an additional 52 weeks and stopped the treatment. A total of 51.6% of patients remained in MMR without treatment reinitiation after 48 weeks of nilotinib cessation. The median duration of nilotinib therapy was 43.5 (32.9–88.9) months in that study. Kimura et al. reported the results of the first-line DADI trial where treatment was stopped for patients who had received first-line dasatinib for at least 36 months with a sustained DMR (defined as ≤ 0.0069%) for at least one year [21]. The TFR at 6 months was 55.2%. The duration of dasatinib treatment and of DMR was 40.4 months (38.1–51.1) and 23.3 months (15.7–29.4), respectively. Although these trials could not demonstrate a clear improvement in TFR rates compared to the discontinuation trials with imatinib [14, 19], they suggested that a shorter treatment period could result in similar TFR rates if 2G-TKIs were used for at least 3 years, and DMR was maintained for at least 1 year. However, the optimal duration of treatment with a 2G-TKI and maintenance of DMR for successful TFR was not clear. This is because the duration of TKI treatment and DMR maintenance in those trials was “at least” and not a fixed duration. Shorter treatment periods for TFR would minimize the problems associated with TKI therapy. We therefore conducted two TKI discontinuation studies in patients with newly diagnosed CML-CP, imposing a fixed DMR duration before cessation of TKI. In the earlier D-News trial, patients with newly diagnosed CML-CP were enrolled and treated with dasatinib for up to 2 years during the induction phase to achieve DMR (defined as 0.0069% or less), then stopped dasatinib treatment after just 2 years of sustained DMR [22]. The median duration of dasatinib treatment before the cessation was 995 days (33.1 months). The 12-month MRFS was 38.5%. In contrast, the estimated MRFS was even lower, at 16.7% at 12 months, in the present D-FREE study. Compared to the D-NewS trial, the duration of the induction phase with dasatinib in the D-FREE study was the same, with a maximum of 2 years, but the duration of DMR was shorter, exactly 1 year. Considering the more stringent definition of DMR (MR4.5) in D-FREE, the decrease in the MRFS rate is presumably due to the shorter duration of TKI treatment, in addition to the shorter DMR duration, although direct comparison between different trials is not possible.

**Table 4:** Published TKI discontinuation clinical trials using second-generation TKIs as first-line treatment

Trial	Number of patients discontinuing TKI	TKI	Study criteria				Total TKI treatment duration, median (months)	DMR duration before TKI discontinuation (months)	TFR/MRFS rate (%); time after discontinuation	References
			Duration of TKI treatment before consolidation phase	Duration of consolidation phase with sustained DMR	TFR eligibility	Definition of relapse for reinitiating TKI				
ENEST freedom	190	nilotinib	At least 2 years	1 year (52 weeks)	MR4.5 for ≥ 1 year (52 weeks)	Loss of MMR	43.5	30.3*^1^	51.6%; 48 weeks	[20]
First-line DADI	58	dasatinib	At least 2 years	1 year	DMR (*BCR-ABL1*^*IS*^ ≤ 0.0069%) for ≥ 1 year	Loss of DMR at two consecutive timepoints or loss of MMR at a single timepoint	40.4	23.3 (median)	55.2%; 6 months	[21]
D-NewS	26	dasatinib	Up to 2 years	2 years	DMR (*BCR-ABL1*^*IS*^ ≤ 0.0069%) for 2 years	Loss of DMR at two consecutive timepoints or loss of MMR at a single timepoint	33.1	24*^2^	38.5%; 1 year	[22]
D-FREE	21	dasatinib	Up to 2 years	1 year	MR4.5 for 1 year	Loss of MR4 at two consecutive timepoints or loss of MMR at a single timepoint	18.9	12.7 (median)	16.7%; 1 year	–

TKI, tyrosine kinase inhibitor; DMR, deep molecular response; TFR, treatment-free remission; MRFS, molecular relapse-free survival; Ref., references; *BCR-ABL1*^*IS*^, *BCR-ABL1* levels on an international scale; MR4.5, *BCR-ABL1*^*IS*^ ≤ 0.0032%; MR4, *BCR-ABL1*^*IS*^ ≤ 0.01%; MMR, major molecular response (*BCR-ABL1*^*IS*^ ≤ 0.1%); –, not applicable

*^1^Calculated from the median time from first achievement of MR4.5 to study entry (18.3 months) and the duration of consolidation phase (52 weeks) specified in the study protocol

*^2^Duration as specified in the study protocol

The European LeukemiaNet (ELN) published a recommendation of requirements for TKI discontinuation, of which the optimal condition was the duration of TKI therapy > 5 years and of DMR > 2 years if MR4.5 [10]. The National Comprehensive Cancer Network (NCCN) guidelines describe criteria for TKI discontinuation as being the duration of approved TKI therapy for at least 3 years and stable MR4 for ≥ 2 years [12], which is less strict than the ELN recommendations. Etienne et al. published the results of their evaluation of the rate of patients eligible for TKI discontinuation and MRFS after stopping according to several recommendations/guidelines, including the ELN and the NCCN [23]. They found that the MRFS of patients who fulfilled the selection criteria proposed by the ELN was significantly different from that of those who did not, whereas no difference in MRFS was observed regarding the criteria proposed by the NCCN. In addition, meeting the selection criteria suggested by the ELN recommendations and front-line 2G-TKIs led to the highest MRFS, reaching 80%, suggesting that TKI therapy for at least 5 years as recommended by the ELN, which is longer than that recommended by the NCCN, may improve TFR. Rea et al. reported on the TFR after two different durations of nilotinib consolidation in patients previously treated with imatinib [24]. They registered the patients who did not achieve MR4.0 after ≥ 24 months of imatinib treatment and treated them with nilotinib. After 1 year of induction, the patients who achieved MR4.0 were randomized to 1 or 2 years of consolidation with nilotinib followed by discontinuation of treatment. The treatment-free survival rate of one and two years of consolidation was 34.5% and 42.5%, respectively, and was not significantly different, suggesting that there is no significant benefit for successful TFR from an additional year of consolidation treatment with nilotinib for patients who achieved sustained DMR after 2 years on nilotinib following a switch from imatinib. It suggests that the duration of DMR may not be the most important factor for the success of TFR. Indeed, the final analysis of the EURO-SKI trial evaluating 755 patients mostly treated with imatinib was recently reported: the prognostic factor for MMR loss after 6 months of TKI discontinuation was the duration of TKI treatment but not the duration of DMR [25]. Although most molecular relapses occurred within 6 months of dasatinib discontinuation in the D-FREE study, the duration of TKI treatment may be important for improving TFR even in patients treated with 2G-TKIs, which are more potent than imatinib. However, there is overlap between the TKI treatment and the DMR periods, making it difficult to analyze which one is more important. The incidence of TKI withdrawal syndrome in this study (2 of 21patiens) was lower than in previous TFR trials (23–30%) [26–28]. This may be related to the short duration of treatment, as a previous study has shown that longer treatment duration predisposes to TKI withdrawal syndrome [28].

It is not known by which mechanism the duration of TKI treatment affects the success of TFR. Previous reports showed that the patients with successful discontinuation had larger and more functional NK cells than the failed patients [29, 30]. Although not examined in our study, it is possible that anti-tumor NK cells may not be able to be maintained by short-term TKI administration. It is also possible that a short TKI treatment period may not reduce CML stem cells to a sufficient level to prevent relapse after treatment cessation. However, the relationship between CML stem cells and TFR is still controversial, as there is a report that CML stem cells can be detected in the peripheral blood of patients during successful TKI discontinuation [31].

Interestingly, one patient maintained TFR for 17.8 months after 18.6 months of dasatinib treatment in our study. It is difficult to detect the difference in patient’s background between successful and unsuccessful TFR in our patients because of its limited number. However, it is very important to find the prognostic factors of successful TFR, so that it may prove possible to shorten duration of TKI treatment in a good prognosis group, thus minimizing AEs, avoiding restriction of pregnancy in women, and reducing the financial challenge of TKI treatment.

This D-FREE study is the largest prospective clinical trial to register newly diagnosed CML-CP patients treated with dasatinib in Japan. In this study, a high MR4.5 attainment rate of 48.8% within 2 years was observed. Although not directly comparable, this rate was higher than that of other trials previously reported worldwide [2, 32, 33]. A similar trend was reported in the Japanese cohort analysis of the DASISION trial [17]. This may be due to the inclusion of a large number of low-risk patients in Japan, where health checkups are well developed and blood cell abnormalities are easily detected at an early stage. Although the study did not focus on collecting safety information during dasatinib treatment, no new serious adverse events were reported. The incidence of cardiovascular events grade 3 or higher during the study period was 1.2%.

In conclusion, DMR for just one year is not enough for successful TFR in CML-CP patients treated with 2G-TKIs for less than three years. A yet unknown sufficient duration of treatment before discontinuation of TKIs is important for improving the TFR treatment goal.

## Data Availability

The data that support the findings of this study are available from the corresponding author, CY, upon reasonable request.
